# Perioperative Anesthetic Management and Early Outcomes Following Primary Total Hip Arthroplasty: A Retrospective Cohort Study in a Moroccan Tertiary Center

**DOI:** 10.7759/cureus.106853

**Published:** 2026-04-11

**Authors:** Othmane Tahri Joutey, Kaoutar Zirhirhi, Anas Mounir, Mohamed Aziz Bouhouri

**Affiliations:** 1 Department of Anesthesiology and Critical Care, Ibn Rochd University Hospital, Casablanca, MAR

**Keywords:** anesthetic techniques, arthroplasty, blood loss, general anesthesia, perioperative management, postoperative complications, retrospective study, spinal anesthesia, total hip arthroplasty, transfusion

## Abstract

Background

Total hip arthroplasty (THA) is a highly effective surgical procedure for advanced hip disease. Perioperative anesthetic management plays a key role in optimizing outcomes, particularly in resource-limited settings where patient characteristics, blood management strategies, and perioperative practices may differ from those reported in high-income countries.

Methods

We conducted a retrospective cohort study including 103 adult patients who underwent primary elective THA at a Moroccan tertiary university hospital between January 2024 and December 2025. Data collected included demographic characteristics, comorbidities, ASA physical status, anesthetic techniques, intraoperative blood loss, transfusion requirements, airway characteristics, and postoperative outcomes. Descriptive statistical analysis was performed.

Results

The cohort was relatively young (mean age 46.4 ± 14.1 years) with a male predominance (sex ratio, 1.5), reflecting a different epidemiological profile compared with Western series. Nearly half of the patients (48.5%) had at least one comorbidity. Spinal anesthesia was the predominant technique (70%). Mean intraoperative blood loss was substantial (800 mL, range 100-1900 mL), with a transfusion rate of 14%. Difficult airway features were mainly observed in patients with underlying rheumatologic conditions. Early postoperative complications were limited, with one surgical site infection. During follow-up, complications occurred in 6.8% of patients, including prosthetic dislocation and aseptic or septic loosening. No mortality was reported.

Conclusion

This study highlights a distinct THA population characterized by younger age and significant perioperative blood loss. Spinal anesthesia was widely used in this cohort and was associated with overall favorable short-term outcomes. Blood management remains a major challenge, particularly in settings where standardized strategies such as systematic tranexamic acid use may not be consistently implemented.

## Introduction

Total hip arthroplasty (THA) is one of the most commonly performed orthopedic procedures worldwide and represents an effective treatment for advanced hip disease, significantly improving pain, mobility, and quality of life [1]. With increasing life expectancy and the growing burden of degenerative and inflammatory joint diseases, the demand for THA continues to rise globally, particularly in high-income countries [2].

Perioperative anesthetic management plays a central role in optimizing surgical outcomes and minimizing complications [3-5]. Several factors may influence perioperative outcomes in THA, including patient comorbidities, preoperative physical status, anesthetic technique, and intraoperative blood loss. Major complications such as thromboembolic events, surgical site infection, and perioperative hemodynamic instability remain important concerns [3-5].

Both general anesthesia and neuraxial techniques are widely used in THA, and each approach has specific advantages and limitations. Several studies have suggested that neuraxial anesthesia may be associated with improved perioperative outcomes in selected patients, although the optimal anesthetic strategy remains a matter of debate [3-5].

Despite advances in anesthetic techniques and perioperative care, THA remains associated with significant challenges, particularly in terms of blood loss, transfusion requirements, and postoperative complications [3-5]. These issues may be even more relevant in resource-variable settings, where patient profiles and perioperative practices may differ from those reported in Western populations.

Data regarding perioperative anesthetic management and outcomes of THA remain limited in North African settings, particularly in Moroccan university hospitals. In addition, specific aspects such as perioperative blood management and airway-related challenges in this population are not well documented.

The aim of this study was to describe anesthetic management and perioperative outcomes in adult patients undergoing primary elective THA at a Moroccan tertiary university hospital.

## Materials and methods

This was a single-center retrospective observational cohort study conducted in the orthopedic and trauma surgery department of Ibn Rochd University Hospital, a tertiary care center in Casablanca, Morocco. The study was carried out over a two-year period, from January 2024 to December 2025.

All consecutive adult patients aged 18 years or older who underwent primary elective THA during the study period were included. A consecutive sampling technique was used. Exclusion criteria were revision arthroplasty, hemiarthroplasty, emergency procedures, and incomplete medical records. A total of 103 patients were included in the study. No formal sample size calculation was performed, as this study was retrospective and descriptive in nature and included all eligible patients during the study period.

Data were retrospectively collected from archived medical records using a standardized data collection form. The variables analyzed included demographic characteristics, comorbidities, American Society of Anesthesiologists (ASA) physical status, anesthetic techniques, airway characteristics, intraoperative blood loss, transfusion requirements, and postoperative outcomes.

Potential confounding factors, including age, sex, comorbidities, ASA physical status, and anesthetic technique, were identified a priori based on their clinical relevance. Given the descriptive nature of the study, no multivariable adjustment was performed; however, these variables were considered during data interpretation.

Preoperative assessment included a detailed medical history, physical examination, and evaluation of comorbidities. Patients were classified according to the ASA physical status classification.

The anesthetic technique was selected based on patient characteristics, comorbidities, ASA physical status, airway assessment, overall clinical condition, and anesthesiologist preference. Spinal anesthesia was performed using hyperbaric bupivacaine (10-15 mg), with intrathecal opioids (fentanyl or morphine) added at the discretion of the attending anesthesiologist based on anticipated surgical duration and patient characteristics. General anesthesia was performed according to standard institutional protocols, including intravenous induction and maintenance with volatile agents.

Standard intraoperative monitoring included electrocardiography, non-invasive blood pressure monitoring, pulse oximetry, and capnography when general anesthesia was used. Intravenous fluid therapy and perioperative blood management were guided by hemodynamic status and estimated blood loss. Perioperative blood management included estimation of intraoperative blood loss and documentation of transfusion requirements. Transfusion decisions were based on clinical judgment and hemoglobin thresholds according to institutional practice, without a formally standardized protocol. Missing data were handled descriptively using available-case analysis.

Postoperative management included multimodal analgesia, antibiotic prophylaxis, and thromboprophylaxis with low-molecular-weight heparin. Postoperative outcomes were assessed during hospitalization and throughout a follow-up period of up to six months after surgery. Follow-up evaluations were based on routine clinical visits and medical record documentation; however, predefined standardized time points were not systematically applied because of the retrospective nature of the study.

Statistical analysis was performed using IBM SPSS Statistics software (version 26; IBM Corp., Armonk, NY, USA). Continuous variables were expressed as mean ± SD or median with range, as appropriate. Categorical variables were presented as frequencies and percentages. Given the descriptive nature of the study, no inferential statistical tests were performed.

The study was conducted in accordance with the principles of the Declaration of Helsinki. Patient data were anonymized before analysis. Given the retrospective nature of the study, the requirement for informed consent was waived. Ethical approval was obtained from the IRB of Ibn Rochd University Hospital.

## Results

A total of 103 patients who underwent primary total hip arthroplasty were included in the study. The mean age was 46.36 ± 14.15 years (range, 18-78), with a male predominance (male-to-female ratio, 1.51:1). Nearly half of the patients (48.5%, n = 50) had at least one comorbidity, most commonly hypertension and diabetes mellitus. According to the ASA classification, the majority of patients were classified as ASA I (66.9%) and ASA II (27.2%), while 5.8% were classified as ASA III. Baseline characteristics are summarized in Table 1.

**Table 1:** Baseline characteristics of patients who underwent primary total hip arthroplasty. ASA: American Society of Anesthesiologists.

Variable	Value
Number of patients	103
Mean age, years	46.36 ± 14.15
Age range, years	18-78
Male	62 (60.2%)
Female	41 (39.8%)
Patients with ≥1 comorbidity	50 (48.5%)
ASA I	69 (66.9%)
ASA II	28 (27.2%)
ASA III	6 (5.8%)

The most common indications for surgery were osteoarthritis, osteonecrosis of the femoral head, and inflammatory joint disease.

Spinal anesthesia was the predominant anesthetic technique, used in 70% of patients (n = 72), while general anesthesia was performed in 30% of cases (n = 31). Among patients who underwent general anesthesia (n = 31), difficult airway was reported in six cases, mainly in patients with ankylosing spondylitis, rheumatoid arthritis, or goiter.

The mean surgical duration was 105 minutes (range, 90-120 minutes). Mean intraoperative blood loss was 800 mL (range, 100-1900 mL), and 14% of patients (n = 14) required blood transfusion. Perioperative anesthetic data and postoperative outcomes are summarized in Table 2.

**Table 2:** Perioperative anesthetic data and postoperative outcomes in patients undergoing primary total hip arthroplasty.

Variable	Value
Spinal anesthesia	72 (70.0%)
General anesthesia	31 (30.0%)
Mean surgical duration, min	105 (90-120)
Mean intraoperative blood loss, mL	800 (100-1900)
Patients requiring transfusion	14 (13.6%)
Difficult airway	6 (5.8%)
Surgical site infection	1 (1.0%)
Prosthetic dislocation	1 (1.0%)
Suspected aseptic loosening	5 (4.9%)
Septic loosening	1 (1.0%)
Death	0 (0%)

Early postoperative complications were rare, with one surgical site infection reported. During follow-up, overall complications occurred in 6.8% of patients (n = 7), including one prosthetic dislocation, five cases of suspected early aseptic loosening, and one case of septic loosening. No thromboembolic events or deaths were reported.

## Discussion

THA is widely recognized as one of the most successful orthopedic procedures in modern medicine [1]. The demand for this procedure is steadily increasing worldwide, reflecting both aging populations and expanding indications [2]. Recent epidemiological data further support this trend, with a continuous rise in the number of primary and revision procedures projected over the coming decades [2].

In the present study, spinal anesthesia was the predominant anesthetic technique, intraoperative blood loss was substantial, and early postoperative outcomes were overall favorable. These observations are consistent with previously reported data in orthopedic surgery, including THA [3-5]. However, our study was purely descriptive and was not designed to compare anesthetic techniques; therefore, no conclusions regarding the comparative effectiveness of anesthetic approaches can be drawn from these data.

A key finding of this study is the relatively young age of the patient population, with a mean age of 46 years. This is lower than that reported in most contemporary series from high-income countries, where patients undergoing primary THA are generally older [6]. This difference may reflect regional epidemiological characteristics and variations in underlying indications, particularly the higher prevalence of osteonecrosis of the femoral head and inflammatory joint diseases in younger populations.

Perioperative blood loss and transfusion requirements remain major concerns in THA [7-9]. In the present study, mean intraoperative blood loss was approximately 800 mL, with a transfusion rate of 14%, findings that are consistent with previously reported data [7-9]. In clinical practice, transfusion strategies are often guided by hemoglobin thresholds and patient comorbidities, particularly cardiovascular risk. Tranexamic acid has been consistently shown to reduce blood loss and transfusion requirements without increasing major complications [7-9]. However, its use was not systematically recorded in our study, limiting assessment of its impact and highlighting an area for improvement in future research.

Enhanced recovery pathways have progressively become central to perioperative care in THA [10,11] and have been associated with improved recovery, reduced complications, and shorter hospital stay [10,11]. Although not specifically analyzed in our study, the overall favorable short-term outcomes observed in our cohort may partly reflect the implementation of standardized perioperative care practices.

Difficult airway was observed mainly in patients with rheumatologic conditions and goiter. This observation is consistent with established airway management guidelines, which emphasize the increased risk of difficult intubation in patients with cervical spine involvement or anatomical distortion [12].

Overall, perioperative outcomes in our cohort were favorable. The incidence of postoperative complications was relatively low and consistent with findings reported in large population-based studies of THA [13]. Several limitations of this study should be acknowledged. The retrospective single-center design limits the generalizability of the findings. The modest sample size and the absence of comparative or adjusted analysis between anesthetic techniques preclude any causal or comparative conclusions. The follow-up duration was limited and non-standardized, which may underestimate the occurrence of long-term complications. Additionally, certain perioperative variables, such as tranexamic acid use, were incompletely documented. The potential for selection bias and residual confounding inherent in retrospective studies should also be considered when interpreting these results.

## Conclusions

This study highlights a distinct population undergoing THA in a Moroccan tertiary center, characterized by relatively young age and significant perioperative blood loss. Spinal anesthesia was the predominant anesthetic technique in this cohort, and overall short-term outcomes were favorable. These findings should be interpreted in a purely descriptive context, as no comparative analysis between anesthetic techniques was performed.

These findings emphasize the importance of adapting perioperative management strategies to population-specific characteristics, particularly in resource-variable settings where patient profiles and clinical practices may differ from those reported in high-income countries. Optimizing blood management strategies, including wider implementation of standardized protocols, may help reduce transfusion requirements and improve perioperative outcomes.

Further prospective studies with larger sample sizes and longer follow-up are needed to better evaluate long-term outcomes and to assess the impact of different anesthetic and blood management strategies in this population. These findings may contribute to improving perioperative management strategies in similar resource-limited settings.
